# Impact of COVID-19 Pandemic on Quality of Life, Anxiety, Connections to Friends, and Access to Resources Among People with HIV: Using the Social Ecological Model

**DOI:** 10.1007/s10461-025-04928-z

**Published:** 2025-10-27

**Authors:** Carol S. Dawson-Rose, Christine Horvat Davey, Emily Huang, Laura Cox, J. Craig Phillips, Motshedisi Sabone, Lufuno Makhado, Emilia Iwu, Kathleen V. Fitch, Sheila Shaibu, Diane Santa Maria, Rebecca Schnall, Panta Apiruknapanond, Tongyao Wang, Álvaro José Sierra Pérez, Tania de Jesús Espinosa, Janessa Broussard, Yvette P. Cuca

**Affiliations:** 1 UCSF School of Nursing, San Francisco, CA USA; 2 International Nursing Network for HIV Research, San Francisco, CA USA; 3 Case Western Reserve University Frances Payne Bolton School of Nursing, Cleveland, OH USA; 4 International Nursing Network for HIV Research, Cleveland, OH USA; 5 University of Ottawa School of Nursing, Ottawa, Canada; 6 International Nursing Network for HIV Research, Ottawa, Canada; 7 International Nursing Network for HIV Research, Gaborone, Botswana; 8 University of Venda, Thohoyandou, South Africa; 9 International Nursing Network for HIV Research, Thohoyandou, South Africa; 10 Rutgers University School of Nursing, Newark, NJ USA; 11 International Nursing Network for HIV Research, Newark, NJ USA; 12 Massachusetts General Hospital, Boston, MA USA; 13 International Nursing Network for HIV Research, Boston, MA USA; 14 Aga Khan University School of Nursing and Midwifery, Nairobi, Kenya; 15 International Nursing Network for HIV Research, Nairobi, Kenya; 16 Cizik School of Nursing at UTHealth, Houston, TX USA; 17 International Nursing Network for HIV Research, Houston, TX USA; 18 Columbia University School of Nursing, New York, NY USA; 19 International Nursing Network for HIV Research, New York, NY USA; 20 Saint Louis College of Nursing, Bangkok, Thailand; 21 International Nursing Network for HIV Research, Bangkok, Thailand; 22 The University of Hong Kong School of Nursing, Pokfulam, Hong Kong; 23 International Nursing Network for HIV Research, Pokfulam, Hong Kong; 24 Promesa, Escuela de Enfermería, Universidad del Valle, Cali, Colombia; 25 International Nursing Network for HIV Research, Cali, Colombia; 26 Yale University School of Nursing, Orange, CT USA; 27 International Nursing Network for HIV Research, Orange, CT USA; 28 UCSF School of Nursing, San Francisco, USA; 29 UCSF School of Nursing, 490 Illinois Street, Floor 9, San Francisco, CA 94143 USA

**Keywords:** HIV, Quality of life, Anxiety, Social connections, Access to resources, COVID-19, Social ecological model

## Abstract

The purpose of this study was to understand the impact of the coronavirus disease 2019 (COVID-19) pandemic and mitigation efforts on health and social outcomes for people with HIV at the individual, social, and structural levels of the Social Ecological Model. The International Nursing Network for HIV collected data for a cross-sectional survey of people with HIV in Botswana, Canada, Colombia, Hong Kong, Kenya, Nigeria, South Africa, Thailand, and the United States from August 2021 through June 2023. Among 1,400 participants, 47.5% experienced decreased quality of life, 40.9% experienced increased anxiety, 33.0% had reduced connection with friends, and 38.8% had reduced access to resources. Participants’ reported impacts of COVID-19 varied by socioeconomic factors. Among these people with HIV, changes in quality of life, anxiety, social connectedness, and access to resources due to the COVID-19 pandemic were significantly associated with individual, social, and structural level factors using the Social Ecological Framework.

## Resumen

El propósito de este estudio fue comprender el impacto de la pandemia por la enfermedad del coronavirus 2019 (COVID-19) y los esfuerzos para mitigar el impacto en las condiciones de salud y sociales de las personas con VIH en los niveles individuales, sociales y estructurales de acuerdo con Modelo Ecológico-Social. La *International Nursing Network for HIV Research* recolectó datos mediante una encuesta de corte transversal aplicada a personas con VIH en Botsuana, Canadá, Colombia, Hong Kong, Kenia, Nigeria, Sudáfrica, Tailandia y los Estados Unidos, entre agosto del 2021 y junio de 2023. De los 1400 participantes el 47.5% experimentó una reducción en su calidad de vida, el 40.9% experimentó un aumento en ansiedad, el 33.0% tuvo una reducción en las relaciones con los amigos y el 38.8% tuvo reducción en el acceso a recursos. El impacto del COVID-19 reportado por los participantes varió según factores socioeconómicos. Entre estas personas con VIH, cambios en la calidad de vida, la ansiedad, la conexión social y el acceso a los recursos durante la pandemia del COVID-19 estuvieron significativamente asociados con factores individuales, sociales y estructurales usados en el Modelo Ecológico Social.

## Background

Social ecological models (SEM) have been widely utilized to enhance our understanding of the global distribution of disease. Health and well-being experiences are contextual and may be influenced by the individual, social/community, and structural factors of SEM [1]. These models are also valuable in designing interventions and policy strategies aimed at preventing illness and promoting health [2]. Social ecological models have previously been applied to examine the experience of people with HIV [3, 4]. COVID-19 disease and the pandemic experience are also well suited for investigation using the Social Ecological Model, particularly in terms of how mitigation strategies affected people with HIV [5, 6]. 

We used the Social Ecological Model (SEM) as a framework to guide our study of the impact of the COVID-19 pandemic and strategies to mitigate its spread on changes in the lives of people with HIV globally [5]. This framework conceptualizes health and behavior as influenced by multiple levels, including the individual, interpersonal and community, and structural levels. Further, the SEM is adaptable, allowing for analysis of how people with HIV navigated pandemic-related changes across various systems and environments, depicting both commonalities and contextual differences across global regions.

### Impacts of COVID-19 at the Individual Level

The COVID-19 pandemic and related protective measures such as shelter-in-place orders, “physical/social distancing,” and waves of infections had substantial impacts at the individual level, including on quality of life, mental health, and substance use [7–10]. A study of 499 people in the Netherlands reported lower health-related quality of life among those living with HIV during the first months of the pandemic compared to those without HIV [11]. Conversely, a global sample of 2,732 men who had sex with men during the early stages of the COVID-19 pandemic showed no difference in quality of life based on HIV status [12]. Others have documented more significant decreases in quality of life among women with HIV compared to men with HIV, yet results remain inconclusive [13]. 

The pandemic also had impacts on mental health. Among 3,810 individuals from Norway, the United States, the United Kingdom, and Australia, participants reported increased levels of emotional distress in the early months of the pandemic [14]. For people with HIV, who already have an increased risk of mental health issues such as anxiety [15], COVID-19 restrictions increased negative mental health symptoms [16]. Among 187 people in St. Louis, United States, increased levels of anxiety and depressive symptoms were higher in people with HIV compared to people without HIV [17]. Among 227 people with HIV in Florida, United States, almost one-third reported declines in mental health due to the pandemic, with reasons ranging from social isolation and increased anxiety to concerns about the disease itself [18]. In China, people with HIV reported a higher incidence of anxiety and depression during the COVID-19 pandemic compared to before the pandemic [19]. 

Mental health and substance use are deeply interconnected, particularly in the context of the COVID-19 pandemic. Studies among men who have sex with men found increases in substance use overall, including binge drinking [20, 21]. Among individuals with both HIV and substance use disorders, the use of illicit substances increased during the pandemic, while confidence in maintaining sobriety declined [22]. Notably, there were significant increases in marijuana use among people with HIV [17]. One study from Miami, United States, indicated that alcohol misuse was more common among individuals without HIV [23]. In contrast, a study conducted in the Netherlands found more significant alcohol use among people with HIV compared to those without HIV [11]. 

### Impacts of COVID-19 at the Social/Community Level

At the social and community level, efforts to mitigate the spread of COVID-19 led to changes in social interactions and feelings of social isolation. In qualitative interviews in New York and California, United States Black and Latino sexual minority men reported social isolation and loss of connection with friends during COVID-19 [24]. In St. Louis, United States, feelings of loneliness were higher in people with HIV compared to people without HIV [17]. Among 273 people with HIV in Miami, United States, women reported greater levels of loneliness than men, and for these women, loneliness was significantly associated with a measure of COVID-19 burden that included disease burden, loss of resources, and disruptions in access to care [25]. In other cases, the pandemic may have had a more positive effect on social support. In a study of 200 young people newly diagnosed with HIV in South Africa, those who were recruited during the COVID-19 pandemic reported significantly higher levels of family support compared to those who were recruited prior to the pandemic, although levels of community support did not differ significantly [26]. 

The pandemic also affected direct social interactions. In two studies of young people in the United States – college students and sexual minority men – participants reported declines in sexual activity during the pandemic lockdowns [27, 28]. In a study of 696 gay and bi-sexual men who have sex with men in the United States, participants reported increases in the number of sexual partners during COVID-19, compared to the three months prior to lockdown [21]. Social distancing policies designed to reduce the spread of COVID-19 led to changes in social connections for people with HIV and those potentially at risk for HIV, with some groups more negatively affected than others.

### Impacts of COVID-19 at the Structural Level

The global pandemic had substantial impacts on HIV at the structural level, particularly on access to resources, health care services, and medications. Data from sexual minority men aged 17–24 in the United States showed that 20% had difficulty accessing pre-exposure prophylaxis (PrEP) prescriptions and other medications, as well as difficulty getting tested for HIV and other sexually transmitted infections (STIs) [28]. For people with HIV, COVID-19 precautions created barriers to access to care, medication, and resources. A systematic review of studies among people with HIV found substantial impacts on access to in-person medical visits and follow-up services, along with reductions in treatment adherence [29]. In one survey of 2,732 men who have sex with men from 103 countries (mostly Brazil, France, Mexico, Russia, and Taiwan), 23% of participants with HIV were unable to access their HIV providers [12], while in another study, 18% had difficulty getting their HIV medications during the pandemic [30]. Rao and colleagues report similar findings in a study of people with HIV in 20 countries [31]. 

The COVID-19 pandemic has been unique in recent history, not only in terms of the disease itself, but also the speed with which it spread, its global impact, and the array of social and structural strategies included in the response. While there are many factors that affect health and well-being among the general population, people with HIV have distinct health needs and, as such, it is important to understand the specific impact of COVID-19 and mitigation strategies on people with HIV globally in order to be better prepared for similar events in the future. While a range of research documents impacts of the pandemic on people with HIV, there is much less research examining changes that people with HIV attribute specifically to the COVID-19 pandemic, both in the United States and globally. Recognizing these changes provides critical insight into how people with HIV perceived pandemic-related challenges and adjusted their health behaviors, which can contribute to tailored interventions that reflect individuals’ lived experiences and interpretations of major societal disruptions. Using the Social Ecological Model can help inform protective strategies for the future.

## Methods

### Study Design

Researchers from the International Nursing Network for HIV Research (the Network) conducted a cross-sectional, international multisite research study to describe the impact of the COVID-19 pandemic on people with HIV [32, 33]. The Coordinating Center at the University of California, San Francisco (UCSF) School of Nursing led the study. All site principal investigators contributed to and approved the study protocol, which was previously published [32]. All participants provided informed consent per local requirements and prior to any research procedures.

### Sample and Recruitment

Participants were recruited from six sites across the United States (Boston, Cleveland, Houston, New York, San Francisco, and San Juan) and sites in Botswana, Canada, Colombia, Hong Kong, Kenya, Nigeria, South Africa, and Thailand [32]. Recruitment was conducted in person and online. In-person recruitment consisted of research staff being present in waiting rooms of HIV clinical and community organizations and passing out flyers about the study. People who were interested could complete study procedures at that time or at a different time, or could contact the research staff via phone to arrange a different time and/or location. Online recruitment included posting information on social media sites and on websites of HIV clinics and community organizations. People who were interested could contact the researchers to be screened for eligibility, which also mitigated the possibility of responses by “bots”. Inclusion criteria were people who self-reported living with HIV and were eligible to provide consent based on age of consent in their location (21 years in San Juan, 18 years in all other sites).

### Procedures

Data were collected from August 2021 through June 2023 via a one-time survey that took approximately 15–45 min to complete based on participant and type of administration (online, telephone, in-person). The study had a prolonged data collection period due to the asynchronous impact of COVID-19 across research sites. Notably, the extended timeline enabled participants to reflect retrospectively on the effects of the pandemic, offering insights into how COVID-19 influenced their lives after its peak. All data were submitted to the Coordinating Center through the Qualtrics study electronic data capture [32]. As the study was conducted internationally, all documentation (i.e., recruitment, consent, and survey materials) was translated into the site’s local language [34]. After completion of the survey, compensation was provided based on the location and ranged from US $2.26 to US $25.

### Measures

The primary outcome of interest – the impact of the COVID-19 pandemic and strategies to mitigate the spread on the lives of people with HIV around the world – was measured using a modified version of the Adolescent Trials Network (ATN) COVID Questionnaire [35]. This survey asks, “Compared to the time before COVID-19, please tell us if COVID-19 and the plans used to manage COVID-19 have impacted you.” The survey consists of seven items regarding general impact (general quality of life, levels of anxiety, quality of sleep, feeling connected to family and friends, and access to resources and internet); four items regarding the impact on income (number of paid work hours, need to support others, difficulty buying food or paying rent); 15 items regarding behaviors and activities (number of sexual partners, opportunities to have sex, use of dating/hook-up apps, access to and use of condoms, access to STI testing or treatment, use of alcohol and drugs, and access to and adherence to hormones for individuals who are transgender); and four items regarding HIV (access and adherence to HIV medications, getting HIV care clinical visits, getting viral loads/labs done). Additionally, the survey assesses loss of job(s), insurance, and housing because of COVID-19.

**Fig. 1:**
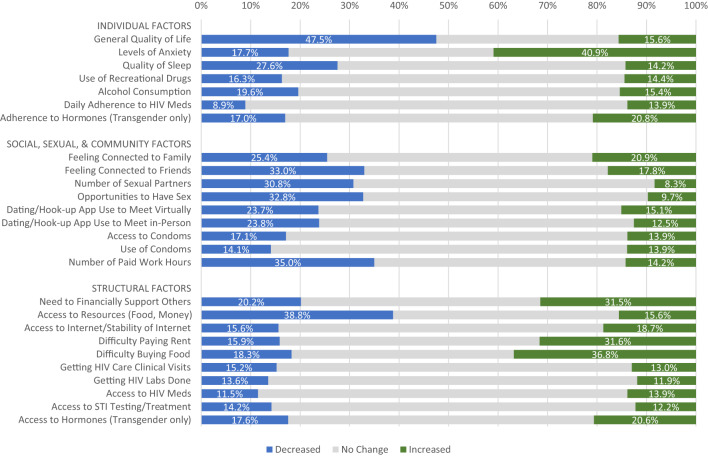
Impact of COVID-19 on Individual, Social, and Structural Factors (*N*=1,400)

For the analysis, these domains were mapped to the three levels of the Social Ecological Model as shown in Fig. 1: individual; social/community factors; and structural factors. Participants responded using a 5-point Likert-type scale with the following responses: (1) Has highly decreased because of COVID-19; (2) Has somewhat decreased because of COVID-19; (3) Has not changed/no different because of COVID-19; (4) Has somewhat increased because of COVID-19; and (5) Has highly increased because of COVID-19. For analysis, response options were collapsed into three categories: highly/somewhat increased, no change/neutral, and highly/somewhat decreased. For each level of the Social Ecological Model, we then identified the ATN COVID Questionnaire outcome with the greatest percentage of negative impact or worsened situation based on Fig. 1. For positive outcomes (e.g., “general quality of life”), worsened situation would include responses of “highly decreased” or “somewhat decreased” because of COVID-19; for negative outcomes, worsened situation would include responses of “highly increased” or “somewhat increased” because of COVID-19.

Independent variables were also organized by the individual, social, and structural levels of the model, and were selected for their potential to be relevant across all contexts:

* Individual factors* include age in years; ethnic minority status (“Do you consider yourself a member of an ethnic or racial minority?” [12]); education level (no schooling; secondary, high school, technical education or less; some university or higher); and alcohol use in the past three months as measured by the ASSIST [36, 37]. 

*Social, Sexual, & Community factors* include social support [38].

*Structural factors* include employment status (currently working, including in the home; not currently working; student); living in stable housing (housing that respondent owns or rents, or in a single room occupancy, and unlikely to lose housing in the next three months); and ability to carry out COVID-19 prevention measures (“To what extent have you been able to carry out these COVID-19 prevention measures in your daily life,” on a 5-point scale of “not at all” to “completely”) [39].

Additional variables were selected for describing the sample:

*Individual factors*: region of the world (Africa, Asia, North America, South America); being of Hispanic origin; being of African origin; living area (urban, suburban, rural); year of HIV diagnosis; having a suppressed HIV viral load (< 50 copies/mL); currently taking HIV medications; Trauma History Screen score [40]; post-traumatic stress disorder (PTSD) [40, 41]; alcohol use (AUDIT-C) [42]; COVID-19 knowledge [43, 44]; fear of COVID-19 [45]; and coping with COVID-19 [46, 47].

*Social, sexual, & community factors*: gender identity; HIV stigma [48]; sexual activity outside the household or with a new partner during the pandemic; and having someone close die from COVID-19.

*Structural factors*: essential worker status during the COVID-19 pandemic; having had at least one HIV primary care visit in the past year; sources of COVID-19 information [43, 44]; and having COVID-19 testing and vaccination available.

### Statistical Analyses

Statistical analyses were completed in Stata 18 [49] and IBM SPSS Statistics (Version 28) [50]. Data were cleaned and descriptive statistics were calculated. Demographic and medical characteristics were summarized as percentages and standard deviations. We identified outcomes from the ATN COVID questionnaire with the greatest levels of a worsened situation/negative impact in each category of the Social Ecological Model, and we included decreased quality of life as an overall measure of pandemic impact. For each of the four outcomes, we conducted a cumulative odds ordinal logistic regression with proportional odds to determine the effect of age, identifying as a racial or ethnic minority (y/n), education (no formal education, secondary or less, some university or higher), employment status (currently working, not currently working, student), stable housing (y/n), ability to carry out COVID-19 prevention measures (y/n), alcohol use (daily alcohol use in the past three months (y/n)), and level of social support on the impact of COVID-19 on the outcome. Statistical significance was set at *p* < 0.05.

## Results

Across all sites, a total of 1,400 people with HIV participated in the study, including 616 (44.0%) from Africa, 511 (36.5%) from North America, 173 (12.4%) from Asia, and 100 (7.1%) from South America (Table 1). Almost one-third (30.1%) reported being a member of a racial or ethnic minority where they live. People identifying as male gender accounted for 51.2% of the sample, 44.4% female, 1.8% transgender, and 1.7% genderqueer. At the time that respondents completed the survey, 51.0% were currently working with 23.1% identifying as essential workers. Only 46.6% were in a stable housing situation. Over half of the study participants never drank alcohol (55.2%); of those who did drink, 52.8% reported binge drinking. The mean social support was moderate at 3.5, and the mean HIV stigma score was 27.8, which indicates a moderate level of stigma. During the pandemic, 25.8% of participants had sex with someone outside their household or with a new partner, possibly exposing them to COVID-19 infection. Although most participants were on antiretroviral medications (90.3%), only 70.1% reported being virally suppressed. 20% of participants lost jobs due to the pandemic.

**Table 1:** Sample characteristics by social ecological model (*N* = 1,400)

	*N* (%)/Mean (SD)
*Individual Characteristics*	
Region	
Africa	616 (44.0%)
Asia	173 (12.4%)
North America	511 (36.5%)
South America	100 (7.1%)
Age	44.8 (SD 13.9) Range: 18–84
Hispanic Origin (yes)	226 (16.1%)
African Origin (yes)	843 (60.2%)
Member of an ethnic or racial minority (yes)	432 (30.1%)
Living Area	
Urban	758 (54.1%)
Suburban	271 (19.4%)
Rural	360 (25.7%)
Education	
No schooling	131 (9.4%)
Secondary, high school, technical education, or less	1,001 (71.5%)
Some university or higher	256 (18.3%)
HIV	
Year of diagnosis (range: 1980–2023) ^a^	2008 (SD 10.0)
Currently on ARV medications (yes)	1,264 (90.3%)
Undetectable viral load (yes)	981 (70.1%)
Trauma History Screen score (possible range: 0–14)	3 (SD 3.4) Range: 0–14
Post-traumatic stress disorder positive screen	138 (9.9%)
How often do you have a drink containing alcohol?	
Never	773 (55.2%)
Monthly or less	325 (23.2%)
2–4 times a month	154 (11.0%)
2–3 times a week	87 (6.2%)
4 or more times a week	51 (3.6%)
Report binge drinking (5 or more drinks on one occasion)	326 (52.8%)
Past 3 months use of substances	
Tobacco	272 (19.4%)
Alcohol	457 (32.6%)
Cannabis	226 (16.1%)
Other illegal or non-prescribed substances	11 (0.8%)
Know how COVID-19 is spread	1,372 (98.0%)
COVID-19 Fear (possible range: 7–35)	20.5 (SD 8.5) Range: 7–35
COVID-19 Coping	
Making time to relax	626 (44.7%)
Healthy behaviors	605 (43.2%)
Breaks from media	523 (37.4%)
Taking care of your body	497 (35.5%)
Connecting with others	489 (34.9%)
Increased news media	367 (26.2%)
Contacting a healthcare provider	293 (20.9%)
Eating more food than usual	213 (15.2%)
Eating high fat or sugary foods	151 (10.8%)
Eating less food than usual	105 (7.5%)
Drinking alcohol	96 (6.9%)
Using cannabis/marijuana	88 (6.3%)
Using prescription drugs (e.g. valium)	64 (4.6%)
Postponing medical care	60 (4.3%)
Over-exercising	36 (2.6%)
Using non-prescription drugs	30 (2.1%)
Cutting or self-injury	25 (1.8%)
Staying Busy	13 (0.9%)
Other	9 (0.6%)
Disconnecting	2 (0.1%)
Social, Sexual, & Community Characteristics	
Gender identity	
Male	717 (51.2%)
Female	621 (44.4%)
Transgender	25 (1.8%)
Genderqueer	24 (1.7%)
Other	10 (0.7%)
Social Support (possible range: 1–5)	3.5 (SD 1.2)
HIV stigma (possible range: 10–50)	27.8 (SD 9.5) Range: 9–50
Sexual activity outside household or with a new partner during COVID-19	361 (25.8%)
Anyone close to you died from COVID-19 (yes)	300 (21.4%)
Structural Characteristics	
Stable Housing (yes)	652 (46.6%)
Employment Status	
Currently working, including in the home	714 (51.0%)
Not currently working	602 (43.0%)
Student	54 (3.9%)
Essential worker	324 (23.1%)
Had at least 1 in-person HIV PCP visit in the past 12 months	1,178 (84.1%)
Had at least 1 virtual HIV PCP visit in the past 12 months	508 (36.3%)
Sources of COVID-19 information	
Local health workers, clinics, and community organizations	727 (51.9%)
Social media	483 (34.5%)
Scientists, doctors, health experts, health organizations	479 (34.2%)
Ordinary people I know personally	408 (29.1%)
Journalists, news organizations, TV news, radio	398 (28.4%)
Government health authorities or other officials	350 (25.0%)
Ordinary people I don’t know personally	171 (12.2%)
Politicians	125 (8.9%)
Other	8 (0.6%)
Employer	6 (0.4%)
General internet	5 (0.4%)
Know where/how to get a COVID-19 test (yes)	1,287 (91.9%)
Availability of COVID-19 vaccination	1,264 (90.3%)
Ability to carry out COVID-19 prevention measures (mostly or completely)	926 (66.1%)
COVID-19 Impacts on Health	
Have lost your job, or one of your jobs (because of COVID-19) (yes)	281 (20.1%)
Have lost your insurance (because of COVID-19) (yes)	106 (7.6%)
Have become homeless or moved in with a friend due to being unable to pay housing costs (because of COVID-19) (yes)	158 (11.3%)
Have had trouble getting HIV medication prescriptions from your doctor because of COVID-19 or the public health efforts to manage it (yes)	157 (11.2%)
Have had trouble getting your HIV medication prescriptions filled at the pharmacy because of COVID-19 or the public health efforts to manage it (yes)	160 (11.4%)
Have had trouble making or keeping your HIV care appointments with your doctor because of COVID-19 or the public health efforts to manage it (yes)	203 (14.5%)

^a^ Data from Hong Kong are excluded because the site did not ask the question in this way

The vast majority of participants could correctly identify how COVID-19 is spread (98.0%), knew how to get tested (91.9%) and had access to vaccination where they lived (90.3%). The mean fear of COVID-19 score was 20.5 (SD 8.5) and ranged from 7 to 35, where higher scores indicate higher levels of fear. Participants used a variety of strategies to cope with the pandemic, the most common being relaxation techniques (44.7%), engaging in healthy behaviors (43.2%), and taking breaks from the news and social media (37.4%). The most common source of COVID-19 information was local health workers, clinics, and community organizations (51.9%); surprisingly, almost equal proportions of participants used social media and scientists, doctors, health experts, and health organizations as sources of information (34.5% and 34.2%).

The COVID-19 pandemic had varied impacts on study participants’ lives (Fig. 1). The Social Ecological Model was used to guide the results of the ATN COVID Questionnaire into individual factors, social/sexual/community factors, and structural factors. For each level of the Social Ecological Model, we then identified the ATN COVID Questionnaire outcome with the greatest percentage of negative impact (“worsened situation”) for further analysis. This approach allowed us to interpret patterns of pandemic related impact through a multilevel lens while maintaining a theoretically grounded model for our analyses: *Individual level* (the outcome with the greatest level of “worsened situation” was increased anxiety); *Social*,* Sexual*,* & Community level* (the outcome with the greatest level of “worsened situation” was decreased feeling connected to friends); *Structural level* (the outcome with the greatest level of “worsened situation” was reduced access to resources). We also examined decreased quality of life as an overall measure of pandemic impact.

Overall quality of life decreased during the pandemic for almost half of the study participants (47.5%). Levels of anxiety increased for 40.9% of participants. Feeling connected to friends decreased for one-third of participants (33.0%). And access to resources decreased for 38.8% of participants. In contrast, the pandemic increased connections to family for 20.9%, and increased access (20.6%) and adherence to (20.8%) hormone therapy for transgender participants.

People with HIV self-identifying as a racial or ethnic minority experiencing decreased ***quality of life*** because of COVID-19 was 0.648, 95% CI [0.461, 0.913] times less than that of people with HIV who did not identify as a minority, a statistically significant effect, Wald *χ*^2^(1) = 6.172, *p* = 0.013 (Table 2). The odds of people with HIV with secondary, high school, tech or less education experiencing decreased quality of life because of COVID-19 was 0.638, 95% CI [0.420, 0.967] times less than that of people with HIV with some university or higher education, a statistically significant effect, Wald *χ*^2^(1) = 4.482, *p* = 0.034. The odds of people with HIV with no formal education experiencing decreased quality of life because of COVID-19 was similar to that of people with HIV with some university or higher education (odds ratio of 0.668, 95% CI [0.219, 2.038]), Wald *χ*^2^(1) = 0.504, *p* = 0.478. The odds of people with HIV with unstable housing experiencing decreased quality of life because of COVID-19 was 1.475, 95% CI [1.028, 2.117] times that of people with HIV with stable housing, a statistically significant effect, Wald *χ*^2^(1) = 4.446, *p* = 0.035. Lower social support was associated with higher odds of considering the COVID-19 pandemic to decrease quality of life, with an odds ratio of 0.811 95% CI [0.704 to 0.933], Wald χ^2^(1) = 8.558, *p* = 0.003.

**Table 2:** Results of ordinal logistic regression analysis predicting likelihood of worse (Lower) quality of life

Variable	Coefficient	Standard Error	Wald χ²	*p*-value	Odds Ratio (95% CI)
Age					
	−0.008	0.006	1.564	0.211	0.992 (0.980, 1.005)
Racial or ethnic minority					
No					(ref)
Yes	**−0.433**	**0.174**	**6.172**	**0.013**	**0.648 (0.461**,** 0.913)**
Education					
No formal education	−0.404	0.570	0.504	0.478	0.668 (0.219, 2.038)
Secondary, tech or less	**−0.450**	**0.213**	**4.482**	**0.034**	**0.638 (0.420**,** 0.967)**
University or higher					(ref)
Employment					
Student					(ref)
Working	−0.044	0.554	0.006	0.936	0.957 (0.323, 2.834)
Not working	0.049	0.544	0.008	0.928	0.952 (0.328, 2.763)
Stable Housing					
Yes					(ref)
No	**0.389**	**0.184**	**4.446**	**0.035**	**1.475 (1.028**,** 2.117)**
Able to carry out COVID-19 measures					
Yes					(ref)
No	−0.221	0.193	1.318	0.251	0.801 (0.549, 1.169)
Daily alcohol (past 3 months)					
Yes	0.107	0.358	0.089	0.766	1.112 (0.552, 2.243)
No					(ref)
Social Support					
	**−0.210**	**0.072**	**8.558**	**0.003**	**0.811 (0.704**,** 0.933)**

Bold text indicates statistical significance *p* < 0.05

The odds of people with HIV with no formal schooling experiencing increased levels of ***anxiety*** because of COVID-19 was 0.233, 95% CI [0.082, 0.664] times less than that of people with HIV with some university or higher education, a significantly significant effect, Wald *χ*^2^(1) = 7.439, *p* = 0.006 (Table 3). The odds of people with HIV with secondary, high school, tech or less education experiencing increased levels of anxiety because of COVID-19 was 0.557, 95% CI [0.372, 0.832] times less than that of people with HIV with some university or higher education, a statistically significant effect, Wald *χ*^2^(1) = 8.143, *p* = 0.004.

**Table 3:** Results of ordinal logistic regression analysis predicting likelihood of worse (Higher) anxiety

Variable	Coefficient	Standard Error	Wald χ²	*p*-value	Odds Ratio (95% CI)
Age					
	0.003	0.006	0.313	0.567	1.003 (0.991, 1.016)
Racial or ethnic minority					
No					(ref)
Yes	0.160	0.172	0.867	0.352	1.173 (0.838, 1.642)
Education					
No formal education	**−1.455**	**0.533**	**7.439**	**0.006**	**0.233 (0.082**,** 0.664)**
Secondary, tech or less	**−0.586**	**0.205**	**8.143**	**0.004**	**0.557 (0.372**,** 0.832)**
University or higher					(ref)
Employment					
Student					(ref)
Working	0.298	0.512	0.338	0.561	1.347 (0.494, 3.675)
Not working	0.389	0.503	0.596	0.440	1.475 (0.550, 3.955)
Stable Housing					
Yes					(ref)
No	0.084	0.173	0.235	0.628	1.087 (0.775, 1.526)
Able to carry out COVID-19 measures					
Yes					(ref)
No	−0.289	0.182	2.511	0.113	0.749 (0.524, 1.071)
Daily alcohol (past 3 months)					
Yes	−0.314	0.356	0.777	0.378	0.731 (0.364, 1.468)
No					(ref)
Social Support					
	−0.095	0.069	1.906	0.167	0.910 (0.795, 1.041)

Bold text indicates statistical significance *p* < 0.05

The odds of people with HIV with unstable housing considering ***feeling connected to friends*** decreased because of COVID-19 was 1.440, 95% CI [1.023, 2.027] times that of people with HIV with stable housing, a statistically significant effect, Wald *χ*^2^(1) = 4.373, *p* = 0.037 (Table 4). Lower social support was associated with higher odds of considering the COVID-19 pandemic to decrease feeling connected to friends, with an odds ratio of 0.754, 95% CI [0.657 to 0.865], Wald χ^2^(1) = 16.221, *p* = 0.001.

**Table 4:** Results of ordinal logistic regression analysis predicting likelihood of worse (Lower) feeling connected to friends

Variable	Coefficient	Standard Error	Wald χ²	*p*-value	Odds Ratio (95% CI)
Age					
	0.004	0.006	0.354	0.552	1.004 (0.992, 1.016)
Racial or ethnic minority					
No					(ref)
Yes	−0.215	0.170	1.597	0.206	0.806 (0.577, 1.126)
Education					
No formal education	−0.919	0.541	2.892	0.089	0.399 (0.138, 1.151)
Secondary, tech or less	−0.294	0.202	2.121	0.145	0.746 (0.502, 1.107)
University or higher					(ref)
Employment					
Student					(ref)
Working	−0.806	0.535	2.266	0.132	0.447 (0.156, 1.276)
Not working	−0.802	0.526	2.323	0.127	0.449 (0.160, 1.258)
Stable Housing					
Yes					(ref)
No	**0.365**	**0.174**	**4.373**	**0.037**	**1.440 (1.023**,** 2.027)**
Able to carry out COVID-19 measures					
Yes					(ref)
No	0.216	0.187	1.326	0.249	1.241 (0.860, 1.790)
Daily alcohol (past 3 months)					
Yes	0.132	0.349	0.143	0.705	1.141 (0.576, 2.259)
No					(ref)
Social Support					
	**−0.283**	**0.070**	**16.221**	**0.001**	**0.754 (0.657**,** 0.865)**

Bold text indicates statistical significance *p* < 0.05

The odds of people with HIV with unstable housing considering ***access to resources*** (food and money) decreased because of COVID-19 was 1.687, 95% CI [1.192, 2.389] times that of people with HIV with stable housing, a statistically significant effect, Wald *χ*^2^(1) = 8.694, *p* = 0.003 (Table 5). Lower social support was associated with higher odds of considering the COVID-19 pandemic to decrease access to resources (food and money), with an odds ratio of 0.817, 95% CI [0.712 to 0.937], Wald χ^2^(1) = 8.379, *p* = 0.004.

**Table 5:** Results of ordinal logistic regression analysis predicting likelihood of worse (Lower) access to resources

Variable	Coefficient	Standard Error	Wald χ²	*p*-value	Odds Ratio (95% CI)
Age					
	−0.011	0.006	3.250	0.071	0.989 (0.977, 1.001)
Racial or ethnic minority					
No					(ref)
Yes	−0.335	0.172	3.801	0.051	0.715 (0.511, 1.002)
Education					
No formal education	−0.221	0.545	0.165	0.684	0.801 (0.276, 2.330)
Secondary, tech or less	0.157	0.201	0.610	0.435	1.170 (0.789, 1.737)
University or higher					(ref)
Employment					
Student					(ref)
Working	0.173	0.515	0.113	0.737	1.189 (0.434, 3.258)
Not working	0.408	0.505	0.652	0.419	1.504 (0.559, 4.049)
Stable Housing					
Yes					(ref)
No	**0.523**	**0.177**	**8.694**	**0.003**	**1.687 (1.192**,** 2.389)**
Able to carry out COVID-19 measures					
Yes					(ref)
No	0.188	0.190	0.977	0.323	1.206 (0.832, 1.750)
Daily alcohol (past 3 months)					
Yes	0.085	0.348	0.060	0.806	1.089 (0.551, 2.152)
No					(ref)
Social Support					
	**−0.202**	**0.070**	**8.379**	**0.004**	**0.817 (0.712**,** 0.937)**

Bold text indicates statistical significance *p* < 0.05

## Discussion

This study used the SEM to guide our analysis of the interactions of people with HIV with their communities and with social and structural agents. This model emphasizes that health and well-being are influenced not just by individual behaviors but also by interpersonal relationships, community environments, and broader structural factors. Our findings support and extend the SEM by demonstrating how factors such as housing instability and social support influence mental health (i.e., anxiety), connections with friends, access to resources, and overall quality of life in people with HIV across the socio-ecological levels during the COVID-19 pandemic.

We examined the impact of the COVID-19 pandemic on a global sample of people with HIV to improve our understanding of how addressing social and structural factors could improve future global health responses to population-level threats. In this study, less than half of the participants had stable housing, only half were currently working, and 20% had lost a job because of COVID-19. Almost one-third of participants described themselves as an ethnic or racial minority; although this is a personal and self-reported characteristic, it exists within power structures that minoritize individuals and reduce economic opportunities and well-being overall. Minoritized identity and racism function as structural factors that influence health and well-being by shaping the systems and policies that govern access to resources, opportunities, and quality of care [51]. Systemic racism embedded in institutions like healthcare, education, housing, and the legal system, creates barriers that lead to disparities in health outcomes. These structural inequities result in poorer health for marginalized groups due to factors such as discriminatory practices, limited economic mobility, and unequal access to healthcare services [52]. Addressing these root causes is essential to achieving health equity.

Almost half of the participants (47.5%) reported decreased general quality of life, 40.9% increased anxiety, 33.0% reduced connections to friends, and 38.8% decreased access to resources because of the COVID-19 pandemic. Similar studies reported that approximately 41% of people with HIV reported increased anxiety during this time [25, 28, 53]. Other literature suggesting that the COVID-19 pandemic exacerbated mental health challenges for this population [18, 28, 54]. We found that these worsened outcomes were associated with individual, social, and structural characteristics.

### Impact of Individual Characteristics

For the purposes of this analysis, racial/ethnic minority status and education level were analyzed as individual characteristics. Study participants who self-reported being a racial or ethnic minority were significantly less likely to report that the pandemic had worsened their quality of life. Prior research among women with HIV before the COVID-19 pandemic showed that women of color reported higher quality of life compared to White women [55], but research in a US nationally representative sample found that Black women had lower health-related quality of life compared to White women [56]. In a study of over 45,000 individuals in Hawaii, United States, White people had lower distress rates than all other minority groups [57]. It may be that minoritized individuals started off the pandemic with a lower quality of life, and because of this, saw less of a decline in quality of life.

Participants with secondary education were less likely to report reduced quality of life compared to those with university or higher education. Those with secondary or less education had reduced odds of reporting worsened anxiety compared to those with more education. This is similar to the findings of a study of 4,479 people living in seven Asian countries in which having a higher level of education was found to be a risk factor for worse mental health during the pandemic [58]. However, our results diverge from prior research, including a study among 904 people in Portugal that found that mid-to-high education levels were associated with better quality of life during the COVID-19 pandemic, and that those with lower education were less likely to report an increase in anxiety [7]. Several factors may explain our results. In the United States, 69% of all immigrants and 74% of undocumented workers are considered essential workers, in contrast to 65% of native-born workers [59]. In turn, these essential workers continued to work during the pandemic. During COVID-19, the United States also implemented a COVID-19 CARES Act Economic Impact Payment. Individuals with lower levels of education were more likely to spend their economic impact payment, while individuals with higher levels of education allocated less of this money for spending needs and debt repayment and saved more of it [60]. Education can play an important role in resource access, particularly during public health emergencies.

### Impact of Social Characteristics

Social support was significantly associated with reduced quality of life, reduced connections to friends, and reduced access to resources. Research conducted after the 2011 tsunami in Japan found that increased social infrastructure resulted in lower mortality in vulnerable people, indicating the importance of community and social infrastructure for preserving health and well-being around public health and environmental emergencies [61]. Additional research shows the importance of social support during times of crisis [62, 63]. Other research has identified pandemic restrictions to stay in one’s home as negatively impacting mental health and social marginalization [64]. The lack of social connections, including opportunities to have sex, has an impact on individuals’ well-being, and social isolation has been associated with adverse HIV outcomes in men who have sex with men [10]. 

### Impact of Structural Characteristics

At the structural level, a lack of stable housing was significantly associated with worsened quality of life, worsened connections to friends, and reduced access to resources. Participants with unstable housing were significantly more likely to report that COVID-19 had worsened their quality of life when compared to people with HIV who reported stable housing, which has been documented in other studies [65]. People without stable housing were also significantly more likely to report that COVID-19 worsened their connections to friends.

Overall, our study reveals the dynamic and interconnected nature of these factors during a crisis. For example, increased anxiety observed among participants can be traced back to disruptions in social and community contexts (e.g., loss of social support), exacerbated by economic instability (e.g., unemployment, unstable housing). These disruptions, in turn, affect access to healthcare and other essential services, creating a cascading effect that worsens health outcomes. Our findings suggest that during a pandemic, the boundaries between social ecological levels become increasingly blurred, and their effects at the individual level are more pronounced. For example, higher levels of education usually correlate with better health outcomes [66, 67]. Our study, however, challenges this assumption by finding that people with HIV with lower levels of education were less likely to report a decrease in quality of life during the pandemic compared to those with higher education. This suggests that in times of crisis, other factors, such as access to stable housing or social support, may play a more critical role in determining health outcomes than education alone. This finding calls for re-evaluating how education is weighted, especially during periods of widespread societal disruption.

During the COVID-19 pandemic, people with HIV faced compounded challenges when experiencing unstable housing. The intersection of unstable housing and HIV created a precarious situation, as the pandemic exacerbated both health and social vulnerabilities [68]. People with HIV, who already manage a chronic condition that requires consistent medical care and medication adherence, found their situation significantly worsened by housing instability. Frequent moves, eviction threats, or homelessness can disrupt access to essential health services, including regular medical check-ups and antiretroviral therapy, increasing the risk of viral suppression failure and overall health deterioration, especially in low- and middle-income countries. Additionally, unstable housing made it challenging to practice social distancing and adhere to public health guidelines, heightening the risk of COVID-19 infection [69]. These challenges underscore the urgent need for targeted support and interventions to ensure that people with HIV have stable housing and continuous healthcare, particularly during global health crises.

### Limitations

The study had several limitations that may affect the generalizability and reliability of its findings. First, using a convenience sample limits the representativeness of the data, as it may not accurately reflect the broader population of people with HIV. Additionally, our data are drawn from individuals and do not include system-level data, which introduces potential biases, as participants’ responses might be influenced by subjective perceptions or recall inaccuracies. Data collection occurred across different sites at various times, including different pandemic periods, and over an extended period of time, which could introduce variability and affect consistency. This variation in context and time, however, provides a global view of the overall impact of the pandemic and mitigation strategies on people with HIV, despite different contexts. In addition, the extended data collection period allowed participants to consider pandemic impacts as a whole, across pandemic peaks and lulls. Given the evolving nature of the COVID-19 pandemic, it is possible that participants’ experiences, stressors, and contextual factors altered over time as public health mandates changed, and communities adapted. This temporal variation represents a limitation and should be considered when interpreting our findings and the generalizability of the results. Finally, while we categorized our data and variables by individual, social, and structural levels, all these data were collected from individuals; no other data sources are included in this analysis.

## Conclusions

In this global sample of diverse people with HIV, less than half reported stable housing or being employed. 20% of participants reported losing employment as a result of the COVID-19 pandemic. One-third of the sample reported being a racial/ethnic minority in their community. The findings highlight the strengths in using social ecological models and identifying the social and structural factors that differentially affect people with HIV at the individual level. These individual level effects include important findings that while the quality of life decreased overall because of COVID-19 and pandemic restrictions, people with HIV with more education or who did not identify as a racial/ethnic minority had worsened quality of life. These findings offer clues across social ecological levels that can help us direct our response to global pandemics in the future, and inform the development of individual-level clinical and social service interventions. These findings also provide evidence to support health and healthcare policy decisions grounded in principles of health equity and human rights necessary for achieving the global health goal of health for all. Addressing the structural inequities experienced by people with HIV is essential to building resilient healthcare systems and ensuring that no population is left behind in future global health responses. By adopting holistic, multilevel strategies that address individual, social, and structural factors, we can work toward achieving sustainable health equity and international Social and Development Goals [70], and improve outcomes for people with HIV worldwide.

## Data Availability

The dataset used and/or analyzed during the current study is available from the corresponding author on reasonable request.
